# In Vitro Evaluation of Antioxidant and Protective Potential of Kombucha-Fermented Black Berry Extracts against H_2_O_2_-Induced Oxidative Stress in Human Skin Cells and Yeast Model

**DOI:** 10.3390/ijms24054388

**Published:** 2023-02-23

**Authors:** Aleksandra Ziemlewska, Martyna Zagórska-Dziok, Zofia Nizioł-Łukaszewska, Patrycja Kielar, Mateusz Mołoń, Dariusz Szczepanek, Ireneusz Sowa, Magdalena Wójciak

**Affiliations:** 1 Department of Technology of Cosmetic and Pharmaceutical Products, Medical College, University of Information Technology and Management in Rzeszow, Sucharskiego 2, 35-225 Rzeszow, Poland; 2 Department of Biology, Institute of Biology and Biotechnology, University of Rzeszów, 35-601 Rzeszów, Poland; 3 Department of Neurosurgery and Paediatric Neurosurgery, Medical University of Lublin, 20-090 Lublin, Poland; 4 Department of Analytical Chemistry, Medical University of Lublin, AlejeRaclawickie 1, 20-059 Lublin, Poland

**Keywords:** *R. nigrum* L., *A. melanocarpa* Michx., *V. myrtillus* L., kombucha, polyphenols, antioxidants, cell skins, *S. cerevisiae*, reactive oxygen species

## Abstract

The fruits of *R. nigrum* L., *A. melanocarpa* Michx., and *V. myrtillus* L. are well-known natural plant materials with proven antioxidant activity. This work attempts to compare the antioxidant properties of extracts of these plants and ferments obtained during their fermentation using a consortium of microorganisms referred to as kombucha. As part of the work, a phytochemical analysis of extracts and ferments was carried out using the UPLC-MS method and the content of the main components was determined. The antioxidant properties of the tested samples and their cytotoxicity were assessed with the use of DPPH and ABTS radicals. The protective effect against hydrogen peroxide-induced oxidative stress was also assessed. The possibility of inhibiting the increase in the intracellular level of reactive oxygen species was carried out on both human skin cells (keratinocytes and fibroblasts) and the yeast *Saccharomyces cerevisiae* (wild-type strains and *sod1Δ* deletion mutants). The conducted analyses showed that the ferments obtained are characterized by a greater variety of biologically active compounds; in most cases they do not cause a cytotoxic effect, show strong antioxidant properties, and can reduce oxidative stress in both human and yeast cells. This effect depends on the concentration used and the fermentation time. The results obtained indicate that the tested ferments can be considered as an extremely valuable raw material protecting cells against the negative effects of oxidative stress.

## 1. Introduction

Skin aging is caused by many mechanisms of internal and external origin. It can be stated that oxidative damage is a very significant cause of skin aging. This is due not only to the production of free radicals, which increases with age, but also to the ability of human skin cells to repair DNA damage [1,2,3]. The impact of oxidative stress on skin aging and the formation of skin disorders is the subject of many scientific studies. It is important to recall that intense exposure to ROS (reactive oxygen species) leads to disorganization of collagen fibers and collagen fragmentation which, in turn, accelerates the skin aging process [4,5]. In addition, the accumulation of ROS leads to structural and functional changes in the skin, including a contribution to the disruption of the extracellular matrix. These changes can also lead to long-term inflammation, as ROS-related cellular damage leads to increased production of proinflammatory cytokines [6,7,8,9].

The proper functioning of the body’s endogenous protective system, which has a significant impact on the function of proteins, the process of cell signaling, and a number of enzyme systems, is very important. The skin contains a pool of endogenous protective antioxidants. It contains enzymatic antioxidants, such as glutathione peroxidase, superoxide dismutase, and catalase, as well as other nonenzymatic substances with antioxidant properties, such as vitamin E, vitamin C, glutathione (GSH), uric acid, and ubiquinol. The epidermis has been shown to contain more antioxidant substances than the dermis [4,5,6]. Studies indicate that it is very important to intensively support the endogenous protective system and to search for effective exogenous antioxidants, including natural plant substances that protect against free radical damage [9,10]. These raw materials include such plants as *Vaccinum myrtillus*, *Ribes nigrum*, and *Aronia melanocarpa*. The analyzed fruits contain numerous phenolic compounds, showing strong antioxidant, antibacterial, or anti-inflammatory activity. In addition, the extracts can regulate oxidation-reduction processes in cells and thus promote the maintenance of redox balance. They possess the ability to inhibit metalloproteinases responsible for the degradation of collagen fibers. *Vaccinum myrtillus* L. extracts have been shown to mitigate the negative effects of sunlight on fibroblasts, which may help improve skin condition and reduce photoaging [11,12,13,14,15,16]. An important aspect, in terms of the proper functioning of the skin’s bacterial microflora, is that plant extracts must be subjected to a fermentation process [17,18]. By subjecting fruit extracts to fermentation, an increase in polyphenol levels was observed in *R. nigrum* and *A. melanocarpa*. [19,20]. The fermented berry fruit extracts studied are additionally characterized by health-promoting properties due to their high content of vitamin C, flavonoids, and anthocyanins compared to nonfermented fruits [19,21]. Studies also show that the fermentation process breaks down larger compounds into phenolic acids, which have antioxidant properties and the ability to scavenge free radicals, associated with greater bioavailability [22].

The role of plant extracts as cosmetic raw materials has been established. However, it is crucial to search for innovative ingredients whose active substances are characterized by greater bioavailability; these may act as high-quality raw materials that can be successfully used in cosmetic products. Therefore, special attention was paid to the fermentation process using a symbiotic culture of bacteria and yeast (SCOBY). While SCOBY was originally used primarily to ferment tea leaves, research is increasingly being undertaken with other raw materials using this consortium of bacteria and yeasts, such as Jerusalem artichoke extract, milk, fresh sweet whey, Coca-Cola, red and white wine, *Echinacea*, Mentha, cherry and grape juice, and others [23,24,25,26]. Importantly, the properties of the resulting ferments depend on the presence of microorganisms and on the plant material being fermented. Therefore, taking into account the proven health-promoting properties of berries, extracts from the tested plants were fermented to compare their biological properties with known extracts. Researchers note that the composition and concentration of metabolites depends on the inoculum source, sugar and tea concentration, fermentation time, and temperature. Proper selection of fermented substrates and fermentation time can help in obtaining innovative cosmetic raw materials [27,28].

In this study, an attempt was made to evaluate the obtained extracts and ferments from fruits of such species as *Vaccinum myrtillus*, *Ribes nigrum*, and *Aronia melanocarpa*. The study determined the content of biologically active compounds in the extracts and ferments. In addition, antioxidant properties were examined and ROS levels were assessed in skin cells exposed to the extracts and kombucha ferments. The experiments were performed on two human cell lines: keratinocytes (HaCaT) and fibroblasts (BJ).

## 2. Results and Discussion

### 2.1. Determination of Bioactive Compounds

Berry fruits are characterized by their high content and wide variety of phenolic compounds. They are diverse in structure and molecular weight, and represented by phenolic acids (benzoic and cinnamic acid derivatives), tannins (gallic and ellagic acid derivatives), and flavonoids such as anthocyanins, flavonols, and flavanols (catechins) [29,30]. Chromatograms of *R. nigrum*, *A. melanocarpa*, and *V. myrtillus* are shown sequentially in the supplementary material in Figures S1–S3. A summary of all identified phenolic compounds for each plant tested, is presented in Table 1. Details of MS identification are presented in Tables S1–S6 in the supplementary material. The profile obtained was similar to those reported in the literature. The content of phenolic compounds in selected berry extracts and their kombucha ferments is shown in Table 2. The results obtained are expressed in µg/mL of extract. The study compared the contents of biologically active compounds in the extract and ferments after 10 and 20 days of fermentation. As can be seen from Table 2, there are clear differences in the content of individual compounds in E, F10, and F20. The differences between fermented and nonfermented extracts may be due to the fact that complex phenolic compounds can be degraded to smaller molecules during the fermentation process [31]. Studies have shown that *A. melanocarpa* fruit is a rich source of hydroxycinnamic acid derivatives. These are mainly represented by caffeic acid derivatives—chlorogenic acid and neochlorogenic acid [32]. They are abundant in the extract (25.16 µg/mL ± 0.21 and 24.9 µg/mL ± 1.09, respectively) as well as in kombucha ferments. Moreover, in the ferments of and *V. myrtillus* fruit, chlorogenic acid is also present in a significant quantity (14.70 µg/mL ± 0.23 for F10 and 17.69 µg/mL ± 0.53 for F20); this value is almost 40 times higher than in its extract.

Anthocyanins occupy an important place in the group of polyphenols found, especially in black berries. The total anthocyanin content of *R. nigrum*, *A. melanocarpa*, and *V. myrtillus* are presented in Table 3. In berries, anthocyanins occur as mono-, di-, or triglycosides, where the glycosidic residues are usually substituted at C3 or, less commonly, at C5 or C7. The most common sugars are glucose, galactose, rhamnose, arabinose, rutinose, sambubiose, and sophorose. Anthocyanin glycoside residues are often acylated by acids: p-coumaric acid, caffeic acid, ferulic acid, and—less frequently—by p-hydroxybenzoic acid, malonic acid, or acetic acid [33]. The results showed that *A. melanocarpa* fruit was the most abundant in anthocyanins. In addition, it was noted that the anthocyanin content of the kombucha ferments from each plant tested was higher than that of the extracts. These differences are greater for F20.

### 2.2. Assessment of Antioxidant Activity

In order to assess the antioxidant potential of the tested extracts and ferments, the DPPH and ABTS assays were used, which allow the assessment of the ability to scavenge free radicals in vitro. The antioxidant activity of the tested samples was expressed as the value of the IC_50_ parameter, which defines the antioxidant concentration causing a 50% decrease in the initial concentration of DPPH and ABTS+ radicals. The antioxidant properties of the extracts and ferments were analyzed in the concentration range from 1 µg/mL to 5000 µg/mL. Ascorbic acid was used as a positive control in a concentration range from 1 µg/mL to 5000 µg/mL. The IC_50_ values obtained for the DPPH and ABTS+ tests are presented in Table 4 and Table 5.

The results indicate that in the DPPH test, the tested extracts and ferments’ antioxidant properties show statistically significant differences. The greatest ability to scavenge the DPPH free radical is shown by extracts and ferments from *A. melanocarpa* followed by *R. nigrum* and *V. myrtillus* (Table 4). In the case of the ABTS+ assay, it was shown that this radical is most strongly scavenged by *A. melanocarpa* followed by *R. nigrum* and *V. myrtillus* (Table 5). In comparison, the IC_50_ value of ascorbic acid was 11.8 µg/mL ± 0.06 in the DPPH assay and 9.3 µg/mL ± 0.04 in the ABTS assay, respectively. The results of both tests clearly indicate that the ferments obtained with the use of kombucha from all three tested plants show stronger antioxidant properties than the extracts. This effect is probably the result of a greater diversity of phenolic compounds and flavonoids in the obtained ferments, the presence of which was shown by chromatographic analyses (Table 1 and Table 2). Stronger antioxidant properties of the analyzed ferments may be due to the formation of such compounds as epigallocatechin, catechin, epicatechin, epigallocatechin gallate, or galloyloquinic after the fermentation process, which are compounds with antioxidant properties proven in numerous works [34,35,36].

**Figure 1:**
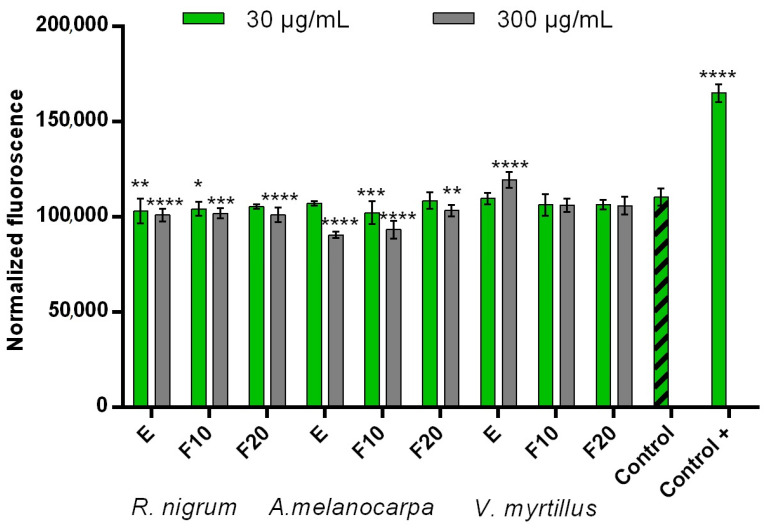
Intracellular ROS levels in HaCaT cells exposed to extracts (E) and ferments (F10 and F20) from *R. nigrum*, *A. melanocarpa*, and *V. myrtillus*. Data are the mean of three independent experiments in which each sample concentration was performed in triplicate. **** *p* < 0.0001, *** *p* < 0.001, ** *p* < 0.01, and * *p* < 0.05 compared to the control.

**Figure 2:**
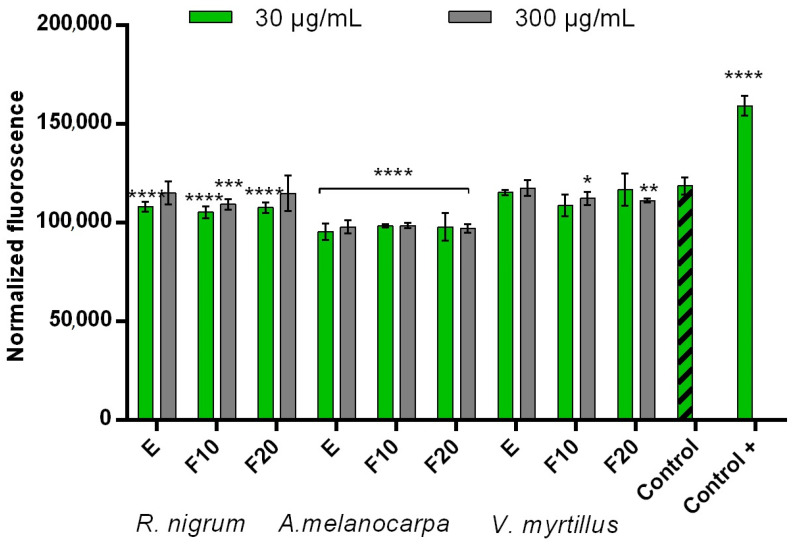
Intracellular ROS levels in BJ cells exposed to extracts (E) and ferments (F10 and F20) from *R. nigrum*, *A. melanocarpa*, and *V. myrtillus*. Data are the mean of three independent experiments in which each sample concentration was performed in triplicate. **** *p* < 0.0001, *** *p* < 0.001, ** *p* < 0.01, and * *p* < 0.05 compared to the control.

In order to assess the possibility of protecting cells against the negative effects of pro-oxidative compounds such as hydrogen peroxide, experiments were carried out on two types of skin cells—human keratinocytes (HaCaT) and fibroblasts (BJ). Due to the fact that oxidative stress has a significant effect on cells, causing the oxidation of lipids, proteins, and DNA, and thus leading to damage to both cells and tissues, compounds that can reduce intracellular levels of reactive oxygen and nitrogen species are very desirable [37]. Toxic products resulting from oxidation processes cause cytostatic effects accompanied by damage to cell membranes and cell death by apoptosis or necrosis [38]. The results of the experiments carried out as part of this work indicate that all extracts and ferments obtained from *R. nigrum*, *A.melanocarpa*, and *V. myrtillus* show cytoprotective effects by reducing the intracellular level of ROS after inducing oxidative stress by 0.1 mM H_2_O_2_ (Figure 1 and Figure 2). This effect was observed in both HaCaT and BJ cells. Both the extract and ferments obtained from *A. melanocarpa* showed particularly strong protective properties, for which the highest decrease in the level of ROS was achieved in both types of cells. Perhaps this is due to the high content of neochlorogenic acid and chlorogenic acid, which have strong antioxidant properties, in this plant [39,40]. However, the total antioxidant properties of the tested samples are certainly related to the interactions between individual phenolic compounds contained in both extracts and ferments. The level of scavenging free radicals depends strictly on the chemical structure of these compounds, various mechanisms of antioxidant activity, reduction potential, and the ability to influence the activity of antioxidant enzymes [41].

**Figure 3:**
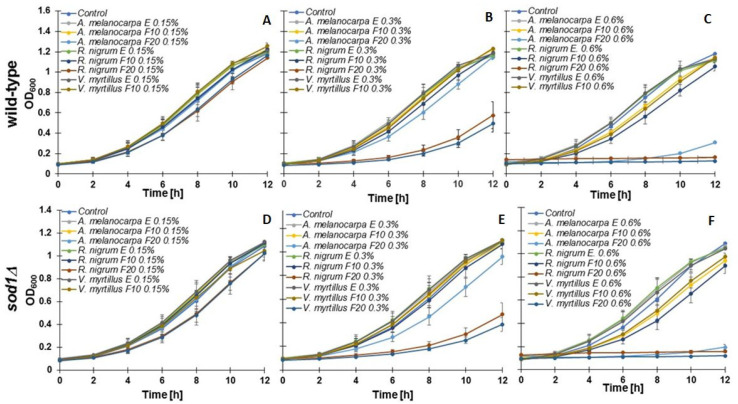
Growth kinetics of the wild-type yeast BY4741 (**A**–**C**) and the isogenic mutant *sod1Δ* (**D**–**F**) treated with extracts and ferments (F10 and F20) of *R. nigrum*, *A. melanocarpa*, and *V. myrtillus*. The optical density (OD_600_) of the culture was measured for 12 h at different points. The data presented are replicates from two independent cultures ± SD, which are smaller or equal in size to the symbol size.

**Figure 4:**
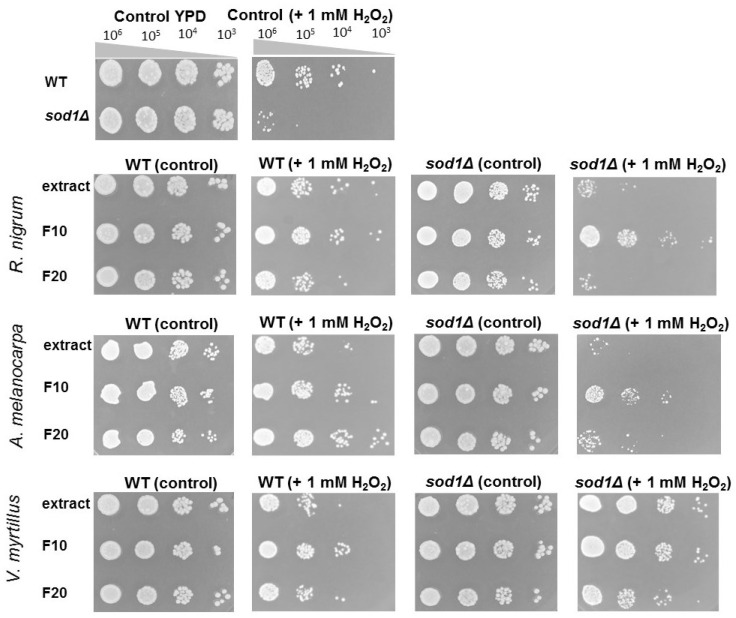
Spot plate assay of the wild-type BY4741 strain (WT) and isogenic mutant *sod1Δ* (*sod1Δ*) treated with extracts and ferments (F10 and F20) of *R. nigrum*, *A. melanocarpa*, and *V. myrtillus* and additionally treated with 1 mM hydrogen peroxide for 1 h (WT+ 1 mM H_2_O_2_ and *sod1Δ* + 1 mM H_2_O_2_). Strains were serially diluted (as indicated (10^6^ to 10^3^). Each spot assay was performed in triplicate.

The budding yeast *Saccharomyces cerevisiae* is a widely used model organism. In this study, the wild-type BY4741 and an isogenic mutant lacking superoxide dismutase 1 (*sod1Δ* strain) were used. Our research has shown that F20 extracts added in small concentration (0.15%) inhibit the cell cycle, especially in the case of *V. myrtillus* and *R. nigrum* ferments (Figure 3A,D). Increasing the concentration of ferments to 0.3% leads to an even more pronounced growth inhibition effect (Figure 3B,E). Interestingly, increasing the ferments’ concentration to 0.6% completely stops cell growth when using *V. myrtillus* and *R. nigrum* F20 ferments. Growth rate was also almost completely stopped when using *A. melanocarpa* F20 in both strains. With the use of ferments F10 (at a concentration 0.6%), a significant growth inhibition was also observed compared to the untreated control (Figure 3C,F). Treatment of cells with extracts had no significant effect on growth rate kinetics. Then, it was asked whether the fermentations and extracts used have the protective ability against hydrogen peroxide, the main precursor of oxygen free radicals in yeast cells. As shown in Figure 4, in the case of the wild-type strain, protective properties were found in *R. nigrum* (F10), *A. melanocarpa* (F10 and F20), and *V. myrtillus* (F10). In the case of the *sod1Δ* mutant, the key contribution of *R. nigrum* F10, *A. melanocarpa* F10, *V. myrtillus* extract, and both types of ferments (F10, F20) in protection against free radicals was observed. The YAP1-GFP construct was used to confirm these observations. The Yap1 transcription factor is concentrated in the nucleus, but only in the positive control. In preincubated cells with extracts or ferments (2 h) subsequently treated with 1 mM hydrogen peroxide (1 h), the transcription factor was located in the cytosol, indicating that oxidative stress was not induced and, thus, the cell protection signaling pathway was not activated (Supplementary Data, Figure S4).

Our research shows that yeast is also useful in studying other organic substances. Recently, we reported on the antioxidant properties of enriched honeys of *A. melanocarpa* L. in vivo using the *S. cerevisiae* model. The modified honey protects yeast cells from oxidative stress caused by H_2_O_2_ when used as a pretreatment agent [42].

### 2.3. Cytotoxicity Assessment

In our study, we evaluated the cytotoxicity of the analyzed kombucha extracts and ferments against skin cell lines—fibroblasts and keratinocytes—in vitro using two types of assays—the neutral red (NR) uptake test and the Alamar Blue (AB) test. The AB assay allows assessment of the viability of the cells under study by evaluating the function of the mitochondrial respiratory chain [43]. The NR test is based on detecting viable cells by assessing the uptake of NR dye, which stains lysosomes in living cells [44]. A cytotoxicity analysis showed that the effect of the tested extract and ferment on skin cell viability in vitro was dependent on both the dose used and duration of the blackberries’ fermentation.

**Figure 5:**
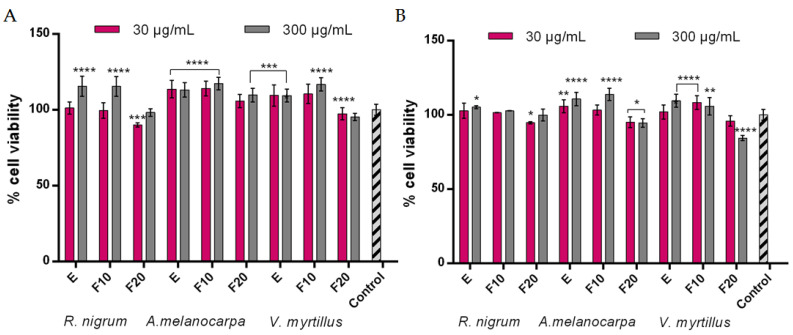
Effect of *R. nigrum, A. melanocarpa*, and *V. myrtillus* extracts and ferments (at the concentration of 30 and 300 μg/mL) on Alamar Blue assay (**A**) and neutral red dye uptake (**B**) in cultured fibroblasts (BJ) after 24 h of exposure. Data are the mean ± SD of three independent experiments, each consisting of three replicates per test group; **** *p* < 0.0001, *** *p*< 0.001, ** *p*< 0.01, and * *p* < 0.05 compared to the control.

**Figure 6:**
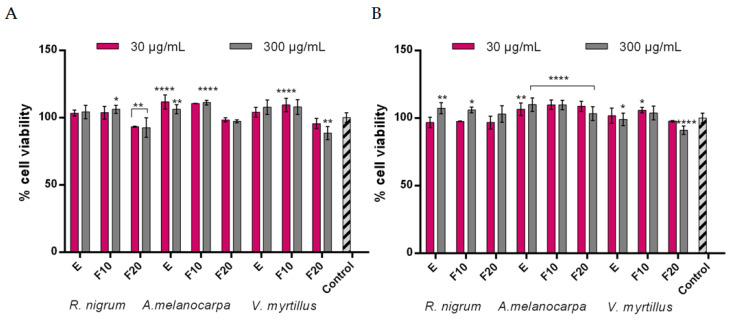
Effect of *R. nigrum, A. melanocarpa*, and *V. myrtillus* extracts and ferments (at the concentration of 30 and 300 μg/mL) on Alamar Blue assay (**A**) and neutral red dye uptake (**B**) in cultured keratinocytes (HaCaT) after 24 h of exposure. Data are the mean ± SD of three independent experiments, each consisting of three replicates per test group; **** *p* < 0.0001, ** *p*< 0.01, and * *p* < 0.05 compared to the control.

Cytotoxicity analysis was performed on *R. nigrum*, *A. melanocarpa*, and *V. myrtillus* fruit extracts and their kombucha ferments after 10 and 20 days of fermentation at concentrations of 30 and 300 µg/mL (Figure 5 and Figure 6). Evaluation of cell viability showed a lack of cytotoxicity against fibroblasts and keratinocytes for the plant extracts tested at all concentrations. Comparing fermentation time, the ferments were found to have a favorable effect on skin cell viability and cell metabolism, using AB and NR assays, after 10 days of fermentation. The percentage of viability of F10-treated cells is comparable or even higher than that of cells treated with the extracts. In the case of keratinocytes, *A. melanocarpa* fruit extract and its ferment showed the highest viability after 10 days of fermentation, reaching about 110% cell viability in both tests. A similar trend was noted for fibroblast cells. In the AB and NR tests, the most favorable effect was obtained for E and F10 from *A. melanocarpa* fruit, achieving 113 and 117% (for the AB test) and 110 and 113% (for the NR test), respectively, at a concentration of 300 µg/mL. Both E and F10 from *R. nigrum* and *V. myrtillus* also showed positive effects on the viability and cellular metabolism of keratinocytes and fibroblasts at all concentrations tested. By increasing the fermentation time to 20 days, a decrease in skin cell viability was observed for all plants tested, with cytotoxicity being greater for samples with the higher concentration of 300 µg/mL. These data correspond with the thesis that long fermentation times may not be beneficial and contribute to the accumulation of harmful products, including organic acids, which can reach levels harmful for direct consumption [27].

Numerous in vitro and in vivo studies indicate that these berries are a rich source of bioactive compounds [45], as confirmed by HPLC-MS (Table 1 and Table 2). In addition to the known health-promoting properties of the plants studied [46,47], the positive effects of berries on skin cells have also been widely studied. The authors confirm the positive effects of berries on UV-B radiation-induced damage to keratinocytes by reducing the levels of reactive oxygen species, inflammatory cytokines, and matrixmetalloproteinase-1 (MMP-1) expression [48]. Exposure to ultraviolet (UV) radiation causes sunburn, inflammation, or photoaging of human skin, which is associated with reduced collagen synthesis. Kim et al. have tested black berries fermented by *Lactobacillus plantarum* on non-human anterior skin fibroblasts (Hs68) and SKH-1 hairless mice. In this study, they showed that black berry bioferment reduces wrinkle formation on dorsal skin and induces epidermal thickening in UVB-irradiated hairless mice [49]. Moreover, the active compounds contained in SCOBY after its fermentation, such as lactic acid and vitamins B3 and C, show protective and antiaging effects via the stimulation of collagen and hyaluronic acid biosynthesis [50].

## 3. Materials and Methods

### 3.1. Plant Material and Fermentation Procedure

Fruits of *R. nigrum*, *A. melanocarpa*, and *V. myrtillus* were collected on controlled and organic plantations. No chemical fertilizers or plant protection products were used in the cultivation. In addition, a preliminary selection was carried out after harvesting the plant material, paying particular attention to chemotaxonomic factors. The kombucha tea fungus starter was purchased from a commercial source from Poland. Initially, the berry extracts were prepared in a sterile beaker by mixing 15 g of fresh fruit and 500 mL of purified water at room temperature. The extracts were obtained by ultrasound-assisted extraction (UAE). The UAE was performed according to the method described by Yang et al. [51]: in an ultrasonic bath (Digital Ultrasonic Cleaner, Berlin, Germany) equipped with a time controller, extracting for 30 min. Then, for fermentation, 50 g of sucrose (final concentration, 10.0% m/v) was added to the resulting extracts and filtered through membrane filters into sterile glass beakers (1000 mL, height 18 cm, diameter 8 cm). Tea mushrooms (3 g) and kombucha (50 mL) were added to the filtrate and fermentation was carried out for 10 and 20 days (in separate beakers) at room temperature (approximately 25 °C). Ferments obtained after 10 days were designated as F10 and those obtained after 20 days were designated as F20.

### 3.2. Determination of Biologically Active Compounds

The main metabolites (phenolic acids and flavonoids) were identified by using an ultra-high performance liquid chromatography (UHPLC) Infinity Series II with a DAD detector and Agilent 6224 ESI/TOF mass detector (Agilent Technologies, Santa Clara, CA, USA). The HPLC conditions were as follows: an RP18 reversed-phase column Titan (Supelco, Sigma-Aldrich, Burlington, MA, USA) (10 cm, 2.1 mm i.d., 1.9 m particle size), a thermostat temperature of 30 C, and a flow rate of 0.2 mL/min. A mixture of water with 0.05% of formic acid (solvent A) and acetonitrile with 0.05% of formic acid (solvent B) was used as a mobile phase. The compounds were separated using gradient elution, according to the following program: 0–9 min from 98% A to 95% A (from 2% to 5% B), 9–24 min from 95% A to 92% A (from 5%to 8% B), 24–45 min from 92% A to 85% A (from 8% to 15% B), and 45–60 min from 85% A to 70% A (from 15% B to 30% B). Chromatograms were recorded from 200 to 400 nm. For the LC–MS analysis, the ion source operating parameters were as follows: drying gas temperature, 325 C; drying gas flow, 8 L/min; nebulizer pressure, 30 psi; capillary voltage, 3500 V; fragmentator, 170 V; and skimmer, 65 V. Ions were acquired in the range of 100 to 1050 m/z.

### 3.3. Total Anthocyanin Content

The total anthocyanin content was evaluated using the pH-differential method, according to the procedure reported by Taghavi et al. [52]. Then, 100 µL of the extract/ferment was mixed with 400 µL of potassium chloride/hydrochloric acid buffer (0.025 M, pH 1.0) and sodium acetate/acetic acid buffer (0.4 M, pH 4.5); after 20 min, the absorbance was measured at 520 and 700 nm. TAC was calculated as o cyanidin 3-O-glucoside equivalent (CG)/100 g of plant material averaged from 3 independent measurements.

### 3.4. Assessment of Antioxidant Activity

#### 3.4.1. DPPH Radical Scavenging Assay

The antioxidant properties of the tested extracts and ferments obtained from *R. nigrum*, *A. melanocarpa*, and *V. myrtillus* were assessed using the DPPH radical (1,1-diphenyl-2-picrylhydrazyl) assay, based on the previously described procedure [53]. Briefly, 4 mM methanolic DPPH solution was added to test samples at concentrations ranging from 1 µg/mL to 5000 µg/mL. Ascorbic acid was used as a positive control in a concentration range from 1 µg/mL to 5000 µg/mL. The samples prepared in this way were thoroughly mixed, and then the absorbance was measured at the wavelength of λ = 517 nm using a UV-VIS spectrophotometer (Thermo Fisher Scientific, Waltham, MA, USA). Water with a DPPH solution was used as a control sample. Three independent experiments, in which each concentration was tested in triplicate, were performed. The percent radical scavenging of DPPH was calculated using the Equation (1). The IC_50_ parameter was then determined, which determines the concentration of the extract or ferment that causes a 50% decrease in the initial concentration of the radical.

(1) $$\%\mathrm{DPPH}\;\mathrm{scavenging}=\frac{\mathrm{Abs}\;\mathrm{control}-\mathrm{Abs}\;\mathrm{sample}}{\mathrm{Abs}\;\mathrm{control}}\times 100$$

where Abs sample is the absorbance of the sample and Abs control is the absorbance of the control sample.

#### 3.4.2. ABTS+ Scavenging Assay

The antioxidant properties of the tested samples of extracts and ferments were also assessed using a solution of 2,2′-azinobis-(3-ethylbenzothiazolin-6-sulfonic acid) (ABTS+) [54]. First, a 7 mM ABTS+ solution and a 2.4 mM potassium persulfate solution were mixed and left for 16 h in the dark at room temperature. This solution was then diluted in methanol to obtain an absorbance of about 1.0 at λ = 734 nm. Then, the test samples (in the concentration range from 1µg/mL to 5000 µg/mL) were mixed with the ABTS+ solution and the absorbance at λ = 734 nm was measured using a UV/VIS spectrophotometer (Thermo Fisher Scientific, Waltham, MA, USA). Ascorbic acid in a concentration range from 1 µg/mL to 5000 µg/mL was used as a positive control. Methanol mixed with ABTS+ was used as a control sample. Three independent experiments, in which each concentration was tested in triplicate, were performed. ABTS+ radical scavenging was calculated from the Equation (2). The IC_50_ parameter was then determined, which determines the concentration of the extract or ferment that causes a 50% decrease in the initial concentration of the radical.

(2) $$\%\;\mathrm{of}\;\mathrm{ABTS}•+\mathrm{scavenging}=1-\frac{\mathrm{As}}{\mathrm{Ac}}\times 100$$

where As is the absorbance of the sample and Ac is the absorbance of the control sample.

#### 3.4.3. Detection of Intracellular Levels of Reactive Oxygen Species (ROS)

In order to determine the protective effect of the analyzed extracts and ferments, their impact on the intracellular level of reactive oxygen species was assessed after exposure of the skin cells to 0.1 mM H_2_O_2_. For this purpose, in vitro studies were carried out using the fluorogenic dye H_2_DCFDA. The determination was made on 2 types of cells—keratinocytes (HaCaT) and fibroblasts (BJ), which were seeded in 96-well plates at a density of 1 × 10^4^ cells per well. After a 24 h incubation of cell cultures with individual concentrations of the analyzed extracts and ferments (30 µg/mL and 300 µg/mL) dissolved in DMEM medium, the medium was aspirated and replaced with 10 µM H_2_DCFDA (Sigma Aldrich, Sant Louis, MO, USA) in serum-free DMEM medium. After 40 min incubation with this cell-permeable probe, HaCaT and BJ cells were treated with 0.1 mM H_2_O_2_. The controls were HaCaT and BJ cells not treated with test compounds, and the positive control was cells treated with only 0.1 mM H_2_O_2_ (without prior exposure to test samples). The 2′,7′-dichlorofluorescein (DCF) fluorescence of individual samples was measured after 60 min at excitation wavelength λ = 485 nm and emission wavelength λ = 530 nm using a microplate reader (FilterMax F5, Thermo Fisher Scientific, Waltham, MA, USA). As part of the work, three independent experiments, in which each sample was tested in triplicate, were performed. The results were compared with cells untreated with the test compounds.

#### 3.4.4. Assessment of Antioxidant Activity Using the *S. cerevisiae* Model

##### Yeast Strains

The strains used in this study were haploid wild-type yeast strain BY4741 (*MATa his3 leu2 met15 ura3*) and isogenic mutant strains *sod1Δ (MATa his3 leu2 met15 ura3 YJR104C::kanMX4)* (*EUROSCARF*) and YAP1-GFP (*MATa his3 leu2 met15 ura3* YML007W-*GFP*) (Life Technologies). 

##### Growth Conditions

Yeast cells were grown in a liquid YPD medium (1% Difco Yeast Extract, 1% Yeast Bacto-Peptone, 2% (w/v) glucose) without (control) or with treatment ferments or extracts on a rotary shaker at 150 rpm, or on a solid YPD medium containing 2% agar. The experiments were carried out at a temperature of 28°C. The data represent the mean values from at least three independent experiments. 

##### Kinetics of Growth Assay

The growth assay was performed on a liquid YPD medium. Yeast cell suspensions were incubated at 28 °C for 12 h with shaking (Heidolph Inkubator 1000 at 1200 rpm). The growth was monitored turbidimetrically in the Anthos 2010 type 17,550 microplate reader at λ = 600 nm by performing measurements at 2 h intervals for 12 h. The data represent the mean values from three independent experiments. 

##### A Spot Test

Growth of the yeast cultures was carried out in YPD medium until exponential phase (OD_600 nm_ between 0.5 and 0.7) and serially diluted according to the indicated concentrations (dilution ratio: 1:10, 1:100, 1:1000, 1:10,000). Five microliters of each cell suspension were spotted onto agar YPD plates. The growth of the cells was measured 48 h after incubation at 28 °C. At least three independent experiments were conducted to confirm each phenotype described in this paper. Spot plate assay was performed using the wild-type BY4741 strain and isogenic mutant *sod1∆* treated with extracts and ferments (F10 and F20) of *R. nigrum*, *A. melanocarpa*, and *V. myrtillus*. Yeast cells were preincubated in extracts or ferments for 2 h. Then, the cells were washed in sterile PBS and treated with 1 mM hydrogen peroxide for 1 h.

##### Localization of the Yap1-GFP Protein in Yeast Cells

The expression of the yeast transcription factor Yap1 in response to oxidative stress was determined as described previously [55]. Ferments or extracts at a concentration of 0.15, 0.3, and 0.6% were added to a yeast cell suspension at a density of 5 × 10^6^ cells/mL and incubated at 28 °C with shaking every 2 h; subsequently, cells were washed twice in PBS, added to hydrogen peroxide at a final concentration of 1 mM, and incubated for 1 h at 28 °C with shaking. Observations of Yap1-GFP localization foci were carried out using an Olympus BX-51 microscope (Tokyo, Japan). Representative results from three independent experiments are shown.

### 3.5. Cytotoxicity Anlysis

#### 3.5.1. Cell Culture

In order to determine the cytotoxic activity of the analyzed extracts and ferments, two types of skin cells were used: fibroblasts (American Type Culture Collection Manassas, VA, USA) and keratinocytes (CLS Cell Lines Service GmbH, Eppelheim, Germany). The cells were grown in Dulbecco’s Modified Eagle’s Medium (DMEM, Biological Industries, Cromwell, CO, USA) with high glucose content (4.5 g/L). The medium was enriched with sodium pyruvate, L-glutamine, 10% fetal bovine serum (Gibco, Waltham, MA, USA), and 1% antibiotics (100 U/mL penicillin and 1000 µg/mL streptomycin, Gibco). The cells were grown in an incubator at 37 °C, in a humidified atmosphere of 95% air and 5% carbon dioxide. After obtaining the required confluence, the medium was removed. The cells were washed with sterile phosphate buffered saline and then detached from the bottom of the culture flasks with trypsin. Next, the cells were placed in fresh medium, plated in 96-well plates, and incubated for 24 h. After this time, the cells were treated with analyzed extracts and kombucha ferments in concentrations of 30 and 300 µg/mL and incubated for another 24 h. The tested formulations containing the extracts and ferments were incubated for 4 h. The control samples were the cells that were not treated with extracts and ferments.

#### 3.5.2. Alamar Blue Assay

The first test used to evaluate the viability of skin cells was Alamar Blue assay according the procedurę of Page et al. [56]. After the incubation of the cells that were treated with the extracts and ferments, the analyzed samples were removed from the wells and then resazurin solution (60 µM) was added. Plates were placed in an incubator for 2 h at 37 °C. Then, fluorescence was measured at wavelength λ = 570 nm using a FilterMax F5 microplate reader (Thermo Fisher Scientific, Waltham, MA, USA).

#### 3.5.3. Neutral Red Uptake Assay

The second test used to evaluate the viability of skin cells was neutral red uptake according the procedure of Borrenfreund et al. [57]. After incubation, the analyzed samples were removed from the wells and then the neutral red dye (40 µg/mL) was added to the wells. Plates were placed in an incubator for 2 h at 37 °C, then the neutral red dye was removed, and the cells were washed with phosphate buffered saline. After this, the phosphate buffered saline was removed and 150 µL of decolorizing buffer was added. The absorbance measurements were performer at wavelength λ = 540 nm using a FilterMax F5 microplate reader (Thermo Fisher Scientific, Waltham, MA, USA).

### 3.6. Statistical Analaysis

The data are presented as means SD of three independent experiments, in which each tested concentration of individual samples was repeated three times; hence, the number “n” from all experiments was nine. The obtained experimental data were analyzed with one-way analysis of variance (ANOVA) followed by Tukey’s multiple comparison test. The statistical significance was determined at **** *p* < 0.0001, *** *p* < 0.001, ** *p* < 0.01, and * *p* < 0.05 compared to the control. The statistical analysis was performed using GraphPad Prism 8.0.1 (GraphPad Software, Inc., San Diego, CA, USA) and Statistica 9.0 (StatSoft, CA, USA).

## 4. Conclusions

The results of the analyses indicate that extracts of *R. nigrum*, *A. melanocarpa*, and *V. myrtillus* and their kombucha ferments are characterized by a significant content of biologically active compounds, such as polyphenolic compounds belonging to phenolic acids (benzoic and cinnamic acid derivatives), tannins (gallic and ellagic acid deisins), and flavonoids (anthocyanins, flavonols, and flavanols responsible for antioxidant activity, among others). In vitro studies using skin cells—fibroblasts and keratinocytes—as well as *S. cerevisiae* yeast have shown that kombucha ferments possess a free radical scavenging capacity after 10 and 20 days. Furthermore, both extracts and ferments have been shown to have a positive effect on skin cell viability and metabolism. The most beneficial effect was observed for F10. Fermented by various microorganisms, such as yeast, many bioactive substances that possess many benefits for humans—not only for health, but also for industry, environment and other aspects—are contained in *Lactobacillus* spp., *Bifidobacterium* spp. bacteria, *Saccharomyces cerevisiae* yeasts, molds, as well as kombucha tea fungi (SCOBY). Nowadays, more and more of these are being produced on an industrial scale and production is becoming more standardized and safer. As the standard of living improves, people are paying increasing attention to health and longevity. The development of fermented plant extracts possesses great potential in the cosmetic industry because, in addition to their probiotic activity supporting beneficial microorganisms inhabiting human skin, they can also constitute a valuable ingredient in pharmaceutical and cosmetic products.

## Figures and Tables

**Table 1:** Bioactive compounds detected using UHPLC/DAD/ESI–MS in *R. nigrum*, *A. melanocarpa,* and *V. myrtillus*. An x indicates the occurrence of the detected compounds in the tested plant.

Molecular Formula	Name of Compound	*R. nigrum*	*A. melanocarpa*	*V.* *myrtillus*
C_6_H_12_O_7_	Gluconic acid	x	x	x
C_7_H_12_O_6_	Quinic acid	x	x	x
C_7_H_6_O_5_	Galic acid	x	x	x
C_14_H_16_O_10_	Galloylquinic acid	x	x	x
C_30_H_26_O_14_	Prodelphinidin B4/B3	x		
C_13_H_16_O_10_	Galloylglucose	x		
C_13_H_16_O_9_	Dihydroxybenzoic acid hexoside	x		x
C_13_H_16_O_8_	Hydroxybenzoic acid hexoside	x		x
C_15_H_14_O_7_	Gallocatechin		x	
C_16_H_18_O_9_	Neochlorogenic acid		x	x
C_15_H_18_O_9_	Caffeoyl hexoside			x
C_15_H_18_O_9_	Caffeoyl glucose	x		
C_15_H_14_O_7_	Epigallocatechin	x	x	x
C_15_H_18_O_8_	Coumaroyl hexoside	x		
C_15_H_14_O_6_	Catechin	x	x	x
C_16_H_18_O_9_	Chlorogenic acid		x	x
C_21_H_20_O_12_	Delphinidin hexoside			x
C_21_H_20_O_11_	Cyanidin 3-glucoside/galactoside		x	
C_27_H_30_ O_16_	Cyanidin 3-sophoroside	x		
C_21_H_20_O_11_	Cyanidin hexoside			x
C_16_H_18_O_9_	Cryptochlorogenic acid			x
C_20_H_18_O_10_	Cyanidin 3-arabinoside		x	x
C_15_H_14_O_6_	Epicatechin	x	x	x
C_27_H_30_O_15_	Cyanidin rutoside	x	x	
C_22_H_18_O_11_	Epigallocatechin gallate	x	x	x
C_20_H_18_O_10_	Cyanidin 3-xyloside		x	
C_7_H_6_O_3_	Salicylic acid	x		
C_27_H_30_O_17_	Quercetin dihexoside		x	
C_27_H_30_O_17_	Myricetin rhamnosylhexoside	x		
C_21_H_20_O_13_	Myricetin 3-O-galactoside	x		x
C_21_H_20_O_12_	Eriodictyol glucuronide		x	x
C_26_H_28_O_16_	Quercetin-3-O-vicianoside		x	
C_27_H_30_O_16_	Quercetin robinobioside		x	
C_27_H_30_O_16_	Quercetin rutoside	x	x	x
C_21_H_20_O_12_	Quercetin glucoside	x	x	x
C_23_H_22_O_13_	Quercetin acetylglucoside	x		
C_27_H_30_O_15_	Kaempferol-3-rutinoside	x	x	
C_21_H_20_O_11_	Kaempferol glucoside	x	x	
C_21_H_20_O_11_	Quercetin rhamnoside			x
C_28_H_32_O_16_	Isorhamnetin rhamnosyl hexoside		x	

**Table 2:** UHPLC/DAD/ESI-MS quantitative analysis of *R. nigrum*, *A. melanocarpa*, and *V. myrtillus* water extracts and kombucha ferments. Values are means ± standard deviation (SD) of triplicate.

Analyzed Plant	Name of Compound	Content (µg/mL)		
		E	F10	F20
*Rubus nigrum*	Gallic acid	0.45 ± 0.02 ^a^	2.46 ± 0.07 ^b^	3.73 ± 0.13 ^c^
	Caffeoyl glucose	1.83 ± 0.08 ^a^	1.36 ± 0.07 ^b^	2.57 ± 0.05 ^c^
	Coumaroyl hexoside	1.01 ± 0.03 ^a^	0.88 ± 0.03 ^b^	1.38 ± 0.05 ^c^
	Salicylic acid	3.27 ± 0.07 ^a^	1.65 ± 0.04 ^b^	1.55 ± 0.05 ^b^
	Dihydroxybenzoic acid hexoside	0.66 ± 0.02 ^a^	0.57 ± 0.00 ^b^	0.59 ± 0.01 ^b^
	Myricetin rhamnosyl hexoside	2.08 ± 0.07 ^a^	1.20 ± 0.01 ^b^	1.51 ± 0.01 ^c^
	Myricetin 3-O-galactoside	2.71 ± 0.02 ^a^	1.41 ± 0.01 ^b^	1.90 ± 0.04 ^c^
	Rutin	3.50 ± 0.18 ^a^	1.92 ± 0.01 ^b^	1.99 ± 0.02 ^b^
	Quercetin glucoside	1.19 ± 0.01 ^a^	0.60 ± 0.02 ^b^	0.69 ± 0.01 ^b^
	Kaempferol-3-rutinoside	0.35 ± 0.03 ^a^	0.21 ± 0.01 ^b^	0.28 ± 0.01 ^a,b^
	Kaempferol glucoside	0.56 ± 0.03 ^a^	0.30 ± 0.05 ^a^	0.42 ± 0.07 ^a^
	Galloyloquinic acid	-	0.73 ± 0.02 ^a^	0.88 ± 0.04 ^a^
	Gallocatechin	1.16 ± 0.04 ^a^	1.59 ± 0.00 ^b^	2.04 ± 0.01 ^c^
	Epigallocatechin	-	3.20 ± 0.09 ^a^	4.37 ± 0.11 ^b^
	Catechin	-	0.27 ± 0.00 ^a^	0.27 ± 0.01 ^a^
	Epicatechin	-	1.56 ± 0.06 ^a^	1.42 ± 0.10 ^a^
	Epigallocatechin gallate	-	4.97 ± 0.20 ^a^	3.18 ± 0.12 ^b^
*Aronia melanocarpa*	Gallic acid	-	1.48 ± 0.04 ^a^	3.19 ± 0.04 ^b^
	Protocatechuic acid	0.13 ± 0.01 ^a^	0.18 ± 0.00 ^b^	0.20 ± 0.00 ^b^
	Neochlorogenic acid	24.9 ± 1.09 ^a^	17.42 ± 0.80 ^b^	21.85 ± 0.33 ^a^
	Chlorogenic acid	25.18 ± 0.21 ^a^	16.80 ± 0.38 ^b^	20.98 ± 0.71 ^c^
	Quercetin rutoside	2.54 ± 0.01 ^a^	1.80 ± 0.06 ^b^	2.31 ± 0.09 ^a^
	Quercetin dihexoside I	0.21 ± 0.01 ^a^	0.16 ± 0.00 ^b^	0.24 ± 0.00 ^c^
	Quercetin dihexoside II	0.09 ± 0.00 ^a^	0.04 ± 0.00 ^a^	0.05 ± 0.00 ^a^
	Quercetin-3-O-vicianoside	0.20 ± 0.01 ^a^	0.23 ± 0.01 ^b^	0.24 ± 0.01 ^b^
	Quercetin robinobioside	0.29 ± 0.00 ^a^	0.14 ± 0.00 ^b^	0.16 ± 0.00 ^b^
	Quercetin glucoside	2.05 ± 0.04 ^a^	0.97 ± 0.02 ^b^	1.22 ± 0.05 ^c^
	Kaempferol-3-rutinoside	0.10 ± 0.00 ^a^	0.26 ± 0.00 ^b^	0.36 ± 0.01 ^c^
	Galloyl quinic		0.93 ± 0.06 ^a^	1.26 ± 0.07 ^b^
	Gallocatechin	-	3.62 ± 0.14 ^a^	4.09 ± 0.17 ^a^
	Catechin	-	1.18 ± 0.01 ^a^	1.47 ± 0.04 ^a^
	Epicatechin	-	1.51 ± 0.33 ^a^	2.58 ± 0.09 ^b^
	Epigallocatechin gallate	-	5.56 ± 0.22 ^a^	7.74 ± 0.02 ^b^
	Epigallocatechin	-	3.66 ± 0.07 ^a^	5.82 ± 0.15 ^b^
*Vaccinium myrtillus*	Gallic acid	-	1.61 ± 0.07 ^a^	2.24 ± 0.09 ^b^
	Galloyl quinic acid	-	0.096 ± 0.01 ^a^	1.28 ± 0.04 ^b^
	Neochlorogenic acid	-	0.18 ± 0.00 ^a^	0.25 ± 0.00 ^b^
	Chlorogenic acid	0.44 ± 0.01 ^a^	14.70 ± 0.23 ^b^	17.69 ± 0.53 ^b^
	Cryptochlorogenic acid	0.24 ± 0.00 ^a^	0.26 ± 0.00 ^a^	0.32 ± 0.01 ^b^
	Dihydroxybenzoic acid hexoside I	0.20 ± 0.00 ^a^	0.95 ± 0.00 ^b^	0.11 ± 0.00 ^c^
	Dihydroxybenzoic acid hexoside II	-	0.10 ± 0.01 ^a^	0.13 ± 0.00 ^b^
	Dihydroxybenzoic acid hexoside III	0.16 ± 0.00 ^a^	0.26 ± 0.01 ^b^	0.33 ± 0.00 ^c^
	Caffeoyl hexoside I	0.16 ± 0.00 ^a^	0.10 ± 0.01 ^b^	0.11 ± 0.00 ^b^
	Caffeoyl hexoside II	0.30 ± 0.01 ^a^	0.30 ± 0.10 ^a,b^	0.37 ± 0.01 ^b^
	Rutoside	0.14 ± 0.00 ^a^	1.09 ± 0.02 ^b^	1.22 ± 0.00 ^b^
	Quercetin glucoside	0.06 ± 0.00 ^a^	0.38 ± 0.01 ^b^	0.37 ± 0.02 ^b^
	Quercetin rhamnoside	0.15 ± 0.00 ^a^	0.10 ± 0.01 ^b^	1.04 ± 0.06 ^b^
	Gallocatechin	-	0.61 ± 0.02 ^a^	0.87 ± 0.03 ^b^
	Epigallocatechin	-	1.11 ± 0.02 ^a^	1.30 ± 0.02 ^a^
	Catechin	-	0.11 ± 0.01 ^a^	0.22 ± 0.01 ^b^
	Epicatechin	-	1.82 ± 0.08 ^a^	2.15 ± 0.03 ^b^
	Epigallocatechin gallate	-	2.76 ± 0.07 ^a^	3.86 ± 0.05 ^b^

The lowercase letters a, b, and c in the same line denote significant differences between the values (*p* ≤ 0.05).

**Table 3:** Total anthocyanin content in *R. nigrum*, *A. melanocarpa*, and *V. myrtillus* extract and kombucha ferments in mg per 100 g of plant material expressed as an equivalent of cyanidin 3-O-glucoside (CG).

	*Ribes nigrum*	*Aronia melanocarpa*	*Vaccinium myrtillus*
Total Anthocyanin Content (mg CG/100 g ± SD)			
E	293.90 ± 2.3 ^a^	410.79 ± 3.5 ^a^	125.24 ± 1.9 ^a^
F10	485.94 ± 5.3 ^b^	713.24 ± 7.8 ^b^	187.03 ± 2.5 ^b^
F20	512.66 ± 4.9 ^c^	873.55 ± 8.6 ^c^	193.53 ± 2.9 ^b^

The lowercase letters a, b, and c in the same column denote significant differences between the values (*p* ≤ 0.05).

**Table 4:** Values of IC50 of DPPH radical scavenging for *R. nigrum*, *A. melanocarpa*, and *V. myrtillus* extract and kombucha ferments after 20 min of exposure. Values are means ± standard deviation (SD) of triplicate.

	*Ribes nigrum*	*Aronia melanocarpa*	*Vaccinium myrtillus*
IC_50_ (µg/mL)			
E	1268 ± 11.2 ^a^	938 ± 6.7 ^a^	4610 ± 17.9 ^a^
F10	753 ± 2.5 ^b^	791 ± 7.4 ^b^	1375 ± 8.5 ^b^
F20	810 ± 4.5 ^c^	722 ± 7.0 ^c^	1739 ± 6.9 ^c^

The lowercase letters, a, b and c in the same column denote significant differences between the values (*p* ≤ 0.05).

**Table 5:** Values of IC_50_ of ABTS+ radical scavenging for *R. nigrum*, *A. melanocarpa*, and *V. myrtillus* extract and kombucha ferments. Values are mean ± standard deviation (SD) of triplicate.

	*Ribes nigrum*	*Aronia melanocarpa*	*Vaccinium myrtillus*
IC_50_ (µg/mL)			
E	219± 2.1 ^a^	151± 3.7 ^a^	313 ± 3.9 ^a^
F10	122 ± 1.9 ^b^	128 ± 2.6 ^b^	131 ± 1.3 ^b^
F20	127 ± 3.3 ^c^	126 ± 2.1 ^c^	130 ± 1.7 ^c^

The lowercase letters a, b, and c in the same column denote significant differences between the values (*p* ≤ 0.05).

## Data Availability

Data are contained within the article.

## Supplementary Materials

The following are available online at: https://www.mdpi.com/article/10.3390/ijms24054388/s1. References [16,58,59,60,61,62,63,64,65,66,67,68] are cited in the supplementary materials.
