# Supplementary material for: In Vitro Evaluation of Antioxidant and Protective Potential of Kombucha-Fermented Black Berry Extracts against H2O2-Induced Oxidative Stress in Human Skin Cells and Yeast Model
Source: Int J Mol Sci. 2023 Feb 23;24(5):4388. doi: 10.3390/ijms24054388 (PMC10002260; doi:10.3390/ijms24054388)
Supplement: Supplementary file 1 [file ijms-24-04388-s001.zip › ijms-2099788-supplementary.pdf]

International Journal of *Molecular Sciences*

Supplementary material

In vitro Evaluation of Antioxidant and Protective Potential of Kombucha-Fermented Black Berry Extracts against H_2O_2-Induced Oxidative Stress in Human Skin Cells and Yeast model

Aleksandra Ziemlewska[1], Martyna Zagórska-Dziok[1], Zofia Nizioł-Łukaszewska[1], Patrycja Kielar[2], Mateusz Mołoń[2], Dariusz Szczepanek[3], Ireneusz Sowa[4],Magdalena Wójciak[4]*

[1] Department of Technology of Cosmetic and Pharmaceutical Products, Medical College, University of Information Technology and Management in Rzeszow, Sucharskiego 2, 35-225 Rzeszow, Poland; aziemlewska@wsiz.edu.pl; mazagorska@wsiz.edu.pl; zniziol@wsiz.edu.pl;

[2] Department of Biology, Institute of Biology and Biotechnology, University of Rzeszów, Rzeszów, Poland; mmolon@ur.edu.pl; kielar.patrycja98@gmail.com

[3] Chair and Department of Neurosurgery and Paediatric Neurosurgery, Medical University of Lublin, 20-090 Lublin, Poland; dariusz.szczepanek@umlub.pl

[4] Department of Analytical Chemistry, Medical University of Lublin, Aleje Raclawickie 1, 20-059 Lublin, Poland; i.sowa@umlub.pl; magdalena.wojciak@umlub.pl

* Correspondence: magdalena.wojciak@umlub.pl

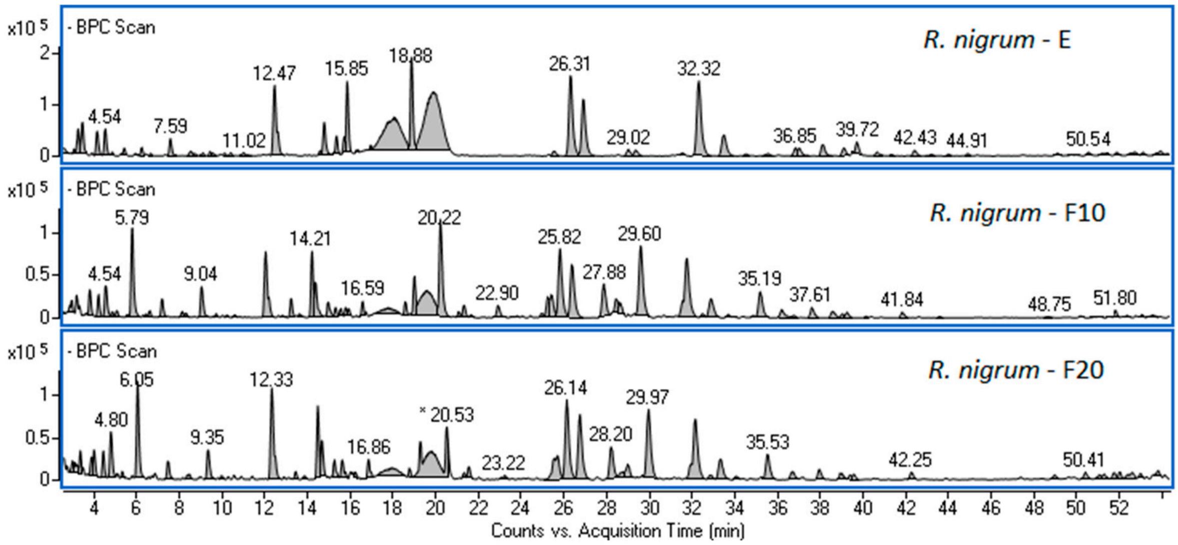

Figure S1. Representative base peak chromatograms (BPC) for *Ribes nigrum* extract and kombucha ferments

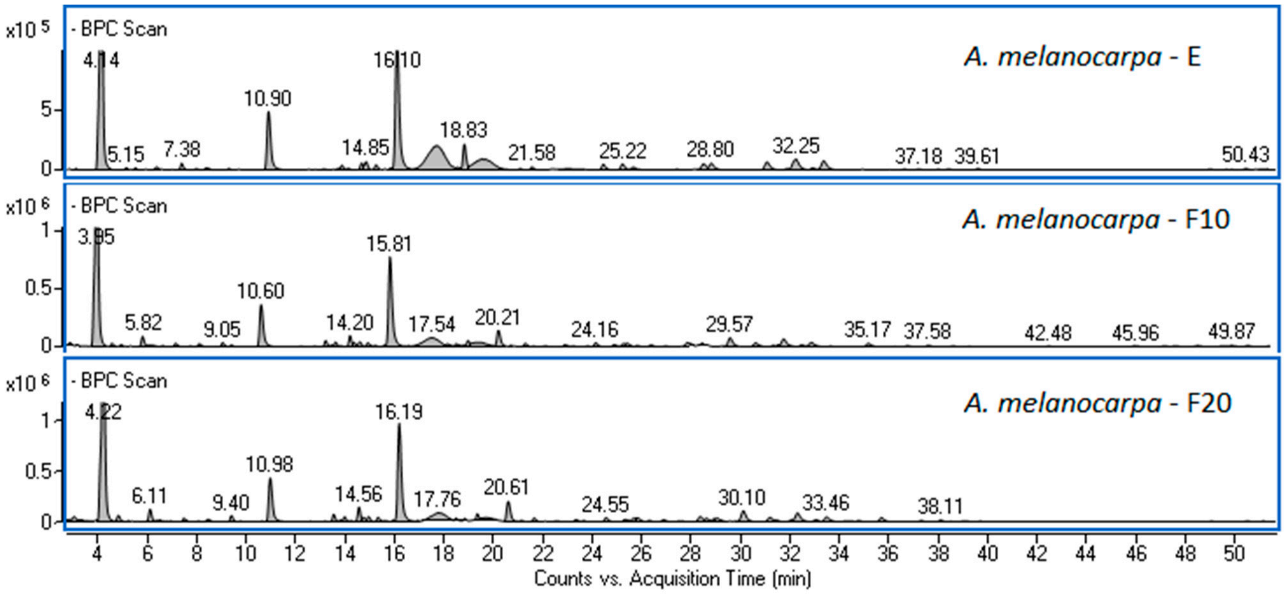

Figure S2. Representative base peak chromatograms (BPC) for *Aronia melanocarpa* extract and kombucha ferments

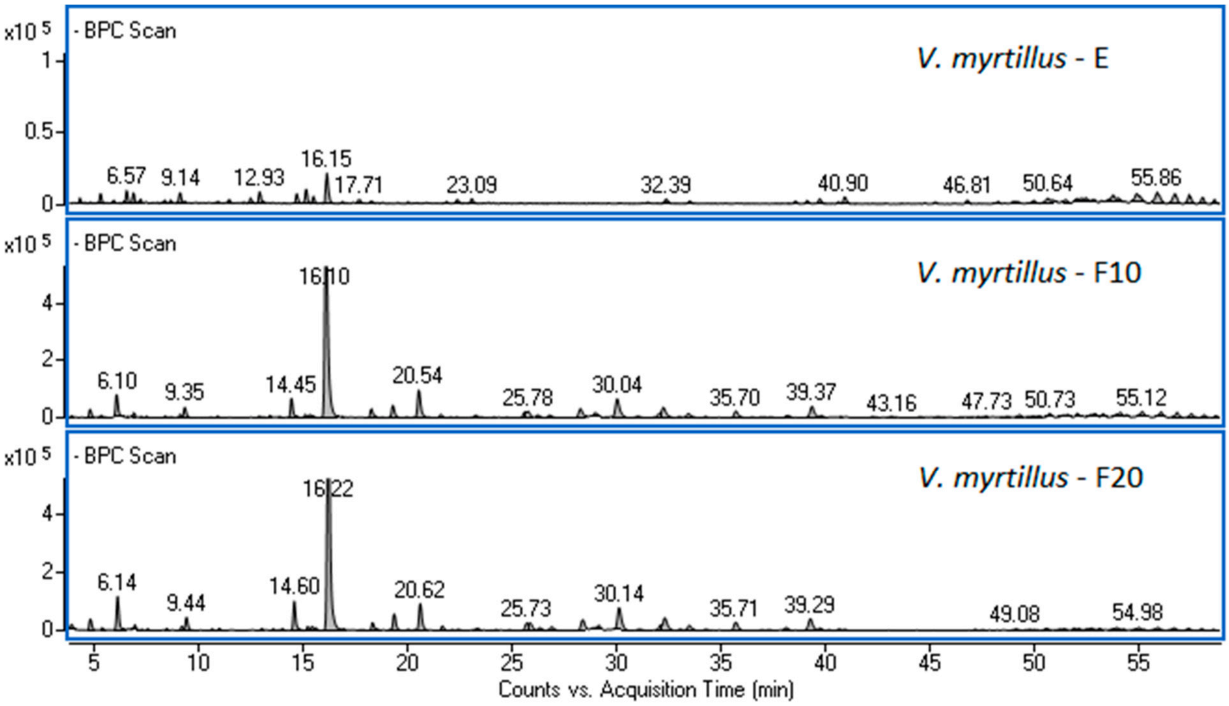

Figure S3. Representative base peak chromatograms (BPC) for *Vaccinum myrtillus* extract and kombucha ferments

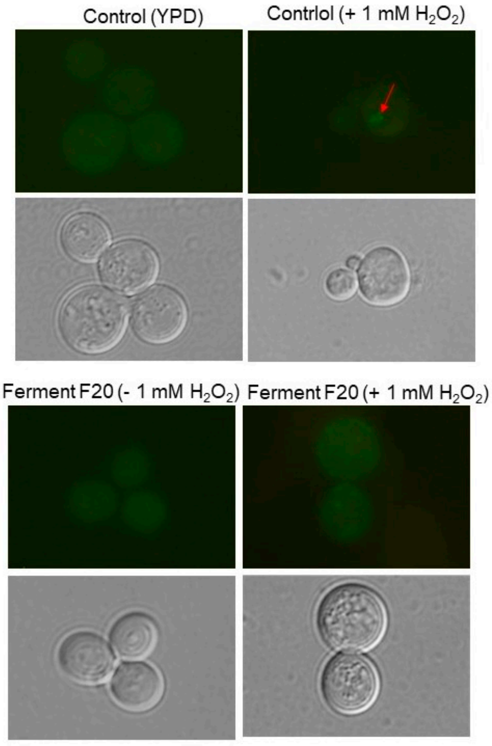

Figure S4. Extracts and ferments protect by Yap1-GFP nuclear localization during oxidative stress inducing by 1 mM hydrogen peroxide. Control - cells from the rich (YPD) culture and 1 mM H2O2 – positive control, cells incubated for 1 h with 1 mM hydrogen peroxide in the YPD medium. The fluorescence images were taken using the Olympus BX-51 microscope equipped with DP-72 digital cameras and cell Sens Dimension software (1000 magnification).Representative results from three independent experiments are shown.

Table S2. Characterization of metabolites identified in *Ribes nigrum* extract by HPLC-MS in negative and positive ion mode

R_T [min]	m/z-H	Error (ppm)	Fragments	Formula	Compound	Identification
Ionization in negative mode						
1.61	191.05652	2.13	-	$C_7H_{12}O_6$	Quinic acid	[1],(std)
4.89	169.01462	2.19	125	$C_7H_6O_5$	Gallic acid	(std)
5.99	609.12617	1.95	-	$C_{30}H_{26}O_{14}$	Prodelphinidin B4/B3	[1]
6.25	331.06842	4.06	125,169	$C_{13}H_{16}O_{10}$	galloylglucose	[1]

7.59	315.07221	0.17	153	$C_{13}H_{16}O_9$	Dihydroxybenzoic acid hexoside	[1,2], *
9.14	299.07799	2.50	137	$C_{13}H_{16}O_8$	Hydroxybenzoic acid hexoside	[2]
9.42	305.06723	1.81	125,169	$C_{15}H_{14}O_7$	Gallocatechin	[2], **
12.47	341.08856	2.20	161,179	$C_{15}H_{18}O_9$	Caffeoylglucose	[1], ***
14.46	305.06711	1.42	125,169	$C_{15}H_{14}O_7$	Epigallocatechin	[3], **
14.78	325.09358	2.11	163	$C_{15}H_{18}O_8$	Coumaroyl hexoside	[2,4], ****
18.05	609.14699	1.44	285	$C_{27}H_{30}O_{16}$	Cyanidin 3-sophoroside	[5]
19.88	593.15301	3.06	285	$C_{27}H_{30}O_{15}$	Cyanidin rutoside	[5]
25.58	137.02498	4.07	-	$C_7H_6O_3$	Salicylic acid	[2],(std)
26.31	625.14321	3.49	316	$C_{27}H_{30}O_{17}$	Myricetin rhamnosylhexoside	[1], *****
26.91	479.08481	3.53	316	$C_{21}H_{20}O_{13}$	Myricetin 3-O-galactoside	[1],(std)
32.32	609.14899	4.72	300,463	$C_{27}H_{30}O_{16}$	Quercetin rutoside	[2],(std)
33.49	463.09001	3.90	300	$C_{21}H_{20}O_{12}$	Quercetin glucoside	(std)
37.09	505.09921	0.88	300	$C_{23}H_{22}O_{13}$	Quercetin acetyl -glucoside	[1]
38.10	593.15211	1.54	285	$C_{27}H_{30}O_{15}$	Kaempferol-3-rutinoside	(std)
39.15	447.09367	0.86	285	$C_{21}H_{20}O_{11}$	Kaempferol glucoside	(std)
Ionization in positive mode						
17.99	611.16133	1.10	287	$C_{27}H_{30}O_{16}$	Cyanidin 3-sophoroside	[5]
19.92	595.16721	2.46	287	$C_{27}H_{30}O_{15}$	Cyanidin rutinoside	[5]

Quantification was based on calibration curves for: *dihydroxybenzoic acid, **catechin, ***caffeic acid, ****p-coumaric acid, *****myricetin rutoside, std - standard

Table S3. Characterization of metabolites identified in *Ribes nigrum* ferment by HPLC-MS in negative ion mode

R_T [min]	m/z-H	Error (ppm)	Fragments	Formula	Compound	Identification
1.59	195.05201	4.99	-	$C_6H_{12}O_7$	Gluconic acid	(std)
4.80	169.01474	2.90	-	$C_7H_6O_5$	Galic acid	(std)
6.05	343.06813	3.08	191	$C_{14}H_{16}O_{10}$	Galloylquinic acid	[3],*
7.52	315.07308	2.92	153	$C_{13}H_{16}O_9$	Dihydroxybenzoic acid hexoside	[1,2],**
9.05	299.07801	2.56	137	$C_{13}H_{16}O_8$	Hydroxybenzoic acid hexoside	[2]
9.38	305.06724	1.84	125,169	$C_{15}H_{14}O_7$	Gallocatechin	[2],***
12.33	341.08836	1.62	161,179	$C_{15}H_{18}O_9$	Caffeoylglucose	[1],****
14.48	305.06741	2.40	125,179	$C_{15}H_{14}O_7$	Epigallocatechin	[3],***
14.70	325.09352	1.93	163	$C_{15}H_{18}O_8$	Coumaroyl hexoside	[2,4],*****
15.33	289.07304	4.41	221,245	$C_{15}H_{14}O_6$	Catechin	(std)
17.98	609.14701	1.48	285	$C_{27}H_{30}O_{16}$	Cyanidin 3-sophoroside	[5]
19.29	289.07214	1.30	221,245	$C_{15}H_{14}O_6$	Epicatechin	(std),***
19.84	593.15319	3.36	285	$C_{27}H_{30}O_{15}$	Cyanidin rutoside	[5]
20.53	457.07969	4.49	125,169,305	$C_{22}H_{18}O_{11}$	Epigallocatechin gallate	[3],***
25.52	137.02507	4.73	-	$C_7H_6O_3$	Salicylic acid	[2], (std)
26.14	625.14115	0.20	316	$C_{27}H_{30}O_{17}$	Myricetin rhamnosylhexoside	[1], ******
26.79	479.08398	1.80	316	$C_{21}H_{20}O_{13}$	Myricetin 3-O-galactoside	[1], (std)
32.15	609.14667	0.92	300,463	$C_{27}H_{30}O_{16}$	Quercetin rutoside	(std)
33.33	463.08999	3.86	300	$C_{21}H_{20}O_{12}$	Quercetin glucoside	(std)
36.73	505.09947	1.39	300	$C_{23}H_{22}O_{13}$	Quercetin acetylglucoside	[1]

37.97	593.15285	2.79	285	$C_{27}H_{30}O_{15}$	Kaempferol-3-rutinoside	(std)
39.06	447.09369	0.90	285	$C_{21}H_{20}O_{11}$	Kaempferol glucoside	(std)

Quantification was based on calibration curve for:*gallic acid, **dihydroxybenzoic acid, ***catechin, ****caffeic acid,*****p-coumaric acid, ******myricetin rutoside, std – standard

Table S3. Characterization of metabolites identified in *Aronia melanocarpa* extract by HPLC-MS in negative and positive ion mode

R_T [min]	m/z-H	Error (ppm)	Fragments	Formula	Compound	Identification
Ionization in negative mode						
1.61	191.05701	4.68	-	$C_7H_{12}O_6$	Quinic acid	(std)
8.42	153.01946	0.83	109	$C_7H_6O_4$	Protocatechuic acid	(std)
10.90	353.08869	2.50	191,135,179	$C_{16}H_{18}O_9$	Neochlorogenic acid	[6–8], (std)
16.10	353.08901	3.40	191,135,179	$C_{16}H_{18}O_9$	Chlorogenic acid	[6–8], (std)
17.68	447.09421	2.06	284	$C_{21}H_{20}O_{11}$	Cyanidin 3-glucoside/galactoside	[5,6]
19.53	417.08434	3.87	284	$C_{20}H_{18}O_{10}$	Cyanidin 3-arabinoside	[5,6,8]
21.56	593.15231	1.88	284	$C_{27}H_{30}O_{15}$	Cyanidin rutoside	[5]
22.99	417.08468	4.69	284	$C_{20}H_{18}O_{10}$	Cyanidin 3-xyloside	[5,6]
25.22	625.14357	4.07	300	$C_{27}H_{30}O_{17}$	Quercetin dihexoside	[6,8],*
25.67	625.14301	3.17	300,563	$C_{27}H_{30}O_{17}$	Quercetin dihexoside	[6,8],*
28.49	463.08841	0.45	287	$C_{21}H_{20}O_{12}$	Eriodictyol glucuronide	[5,8]
28.80	595.13251	3.44	409	$C_{26}H_{28}O_{16}$	Quercetin-3-O-vicianoside	[6–8],*
31.07	609.14899	4.72	300	$C_{27}H_{30}O_{16}$	Quercetin robinobioside	[5,8]*
32.25	609.14862	4.12	300,463	$C_{27}H_{30}O_{16}$	Quercetin rutoside	[6–8](std)
33.37	463.08991	3.69	300	$C_{21}H_{20}O_{12}$	Quercetin glucoside	[6–8],(std)
38.10	593.15299	3.02	285	$C_{27}H_{30}O_{15}$	Kaempferol-3-rutinoside	[6],(std)
39.63	623.16456	4.49	314	$C_{28}H_{32}O_{16}$	Isorhamnetin rhamnosyl - hexoside	[6–8]

Ionization in positive mode						
17.85	449.11001	4.85	287	$C_{21}H_{20}O_{11}$	Cyanidin 3-glucoside/galactoside	[5,6,8]
19.53	419.09911	4.39	287	$C_{20}H_{18}O_{10}$	Cyanidin 3-arabinoside	[5,6,8]
21.56	595.16611	0.61	287	$C_{27}H_{30}O_{15}$	Cyanidin rutinoside	[5]
22.99	419.09871	3.44	287	$C_{20}H_{18}O_{10}$	Cyanidin 3-xyloside	[5,6,8]

*Quantification was based on calibration curve for quercetin rutoside, std- standard

Table S4. Characterization of metabolites identified in *Aronia melanocarpa* ferment by HPLC-MS in negative ion mode

R_T [min]	m/z-H	Error (ppm)	Fragments	Formula	Compound	Identification
1.59	195.05132	1.50	-	$C_6H_{12}O_7$	Gluconic acid	(std)
4.83	169.01501	4.49	-	$C_7H_6O_5$	Galic acid	(std)
6.11	343.06871	4.77	191	$C_{14}H_{16}O_{10}$	Galloylquinic acid	[3],*
8.45	153.01998	4.21	109	$C_7H_6O_4$	Protocatechuic acid	(std)
9.40	305.06788	3.93	125,169	$C_{15}H_{14}O_7$	Gallocatechin	[3],**
10.98	353.08881	2.84	191,135,179	$C_{16}H_{18}O_9$	Neochlorogenic acid	[6–8](std)
14.56	305.06791	4.03	125,169	$C_{15}H_{14}O_7$	Epigallocatechin	[3],**
15.46	289.07201	0.86	221,245	$C_{15}H_{14}O_6$	Catechin	[8],(std)
16.19	353.08921	3.97	191,135,179	$C_{16}H_{18}O_9$	Chlorogenicacid	[6–8](std)
17.76	447.09504	3.92	284	$C_{21}H_{20}O_{11}$	Cyanidin 3-glucoside/galactoside	[5,6,8]
19.36	289.07223	1.61	221,245	$C_{15}H_{14}O_6$	Epicatechin	[3,8],(std)
19.74	417.08412	3.35	284	$C_{20}H_{18}O_{10}$	Cyanidin 3-arabino-side	[5,6,8]
20.61	457.07899	2.96	125,169,305	$C_{22}H_{18}O_{11}$	Epigallocatechingallate	[3],**
21.65	593.15298	3.01	284	$C_{27}H_{30}O_{15}$	Cyanidin rutoside	[5]
23.01	417.08452	4.30	284	$C_{20}H_{18}O_{10}$	Cyanidin 3-xyloside	[5,6,8]

25.32	625.14322	3.51	300	$C_{27}H_{30}O_{17}$	Quercetin dihexoside	[6,8],***
25.74	625.14401	4.77	300	$C_{27}H_{30}O_{17}$	Quercetin dihexoside	[6,8],***
28.49	463.08865	0.97	287	$C_{21}H_{20}O_{12}$	Eriodictyol glucuronide	[5,8]
31.20	609.14862	4.12	300	$C_{27}H_{30}O_{16}$	Quercetin robinobioside	[5,8],***
32.28	609.14833	3.64	300,463	$C_{27}H_{30}O_{16}$	Quercetin rutoside	[6–8],(std)
33.46	463.09001	3.90	300	$C_{21}H_{20}O_{12}$	Quercetin glucoside	[6–8],(std)
38.10	593.15321	3.39	285	$C_{27}H_{30}O_{15}$	Kaempferol-3-rutinoside	(std)
39.13	447.09401	1.62	284	$C_{21}H_{20}O_{11}$	Kaempferolglucoside	[6],(std)
39.73	623.16421	3.93	314	$C_{28}H_{32}O_{16}$	Isorhamnetin rhamnosylhexoside	[6–8]

*Quantification was based on calibration curve for gallic acid,**Quantification was based on calibration curve for catechin, ***Quantification was based on calibration curve for quercetin rutoside , std – standard

Table S5. Characterization of metabolites identified in *Vaccinium myrtillus* extract by HPLC-MS in negative and positive ion mode

R_T [min]	m/z-H	Error (ppm)	Fragments	Formula	Compound	Identification
Ionization in negative mode						
1.60	191.05661	2.59	-	$C_7H_{12}O_6$	Quinic acid	(std)
6.57	315.07301	4.90	153,109	$C_{13}H_{16}O_9$	Dihydroxybenzoic acid hexoside	[9,10],*
7.55	315.07299	2.64	153	$C_{13}H_{16}O_9$	Dihydroxybenzoic acid hexoside	[9,10],*
9.14	315.07312	3.05	153	$C_{13}H_{16}O_9$	Dihydroxybenzoic acid hexoside	[10],*
12.93	341.08881	2.94	179,135	$C_{15}H_{18}O_9$	Caffeoylhexoside	[10],**

15.15	341.08899	3.46	179,135	$C_{15}H_{18}O_9$	Caffeoylhexoside	[10],**
16.22	353.08845	1.82	191,179	$C_{16}H_{18}O_9$	Chlorogenic acid	[10],(std)
16.60	463.08857	0.81	301	$C_{21}H_{20}O_{12}$	Delphinidin hexoside	[11,12]
17.85	447.09354	0.57	285	$C_{21}H_{20}O_{11}$	Cyanidinhexoside	[11,12]
18.32	353.08891	3.12	179,191	$C_{16}H_{18}O_9$	Cryptochlorogenic acid	[10], (std)
19.17	417.08299	0.64	285	$C_{20}H_{18}O_{10}$	Cyanidin-3-arabinoside	[11,12]
32.39	609.14887	4.53	300,463	$C_{27}H_{30}O_{16}$	Quercetin rutoside	[10], (std)
33.50	463.09052	4.99	300	$C_{21}H_{20}O_{12}$	Quercetin glucoside	[10], (std)
39.31	447.09544	4.81	300	$C_{21}H_{20}O_{11}$	Quercetin rhamnoside	[10], (std)
Ionization in positive mode						
16.61	465.10285	0.21	303	$C_{21}H_{20}O_{12}$	Delphinidin hexoside	[11,12]
17.87	449.10789	0.12	287	$C_{21}H_{20}O_{11}$	Cyanidin hexoside	[11,12]
19.21	419.09737	0.23	287	$C_{20}H_{18}O_{10}$	Cyanidin-3-arabinoside	[11,12]

Quantification was based on calibration curves for: *dihydroxybenzoic acid, **caffeic acid, std – standard

Table S6. Characterization of metabolites identified in *Vaccinium myrtillus* ferment by HPLC-MS in negative ion mode

R_T [min]	m/z-H	Error (ppm)	Fragments	Formula	Compound	Identification
1.58	195.05189	4.41	-	$C_6H_{12}O_7$	Gluconic acid	(std)
4.84	169.01488	3.72	-	$C_7H_6O_5$	Galic acid	(std)
6.14	343.06850	4.16	191	$C_{14}H_{16}O_{10}$	Galloylquinic acid	[3],*
6.56	315.07291	2.39	153,109	$C_{13}H_{16}O_9$	Dihydroxybenzoic acid hexoside	[9],**
7.57	315.07287	2.26	153	$C_{13}H_{16}O_9$	Dihydroxybenzoic acid hexoside	[9],**
9.21	315.07300	2.67	153	$C_{13}H_{16}O_9$	Dihydroxybenzoic acid hexoside	[10],**
9.44	305.06793	4.10	153,169	$C_{15}H_{14}O_7$	Gallocatechin	[2],***
11.00	353.08898	3.32	191,179	$C_{16}H_{18}O_9$	Neochlorogenic acid	[10]

13.02	341.08896	3.37	179,135	$C_{15}H_{18}O_9$	Caffeoylhexoside	[10],****
14.60	305.06796	4.19	125,169	$C_{15}H_{14}O_7$	Epigallocatechin	[3],***
15.24	341.08881	2.94	179,135	$C_{15}H_{18}O_9$	Caffeoylhexoside	[10],****
15.47	289.07287	3.82	221,245	$C_{15}H_{14}O_6$	Catechin	[10], (std)
16.22	353.08892	3.15	191,179	$C_{16}H_{18}O_9$	Chlorogenic acid	[10]
16.65	463.08941	2.61	301	$C_{21}H_{20}O_{12}$	Delphinidin hexoside	[11,12]
17.80	447.09391	1.39	285	$C_{21}H_{20}O_{11}$	Cyanidin hexoside	[11,12],
18.35	353.08884	2.92	191,179	$C_{16}H_{18}O_9$	Cryptochlorogenic acid	[10]
19.20	417.08375	2.46	285	$C_{20}H_{18}O_{10}$	Cyanidin- 3-arabinoside	[11,12]
19.37	289.07298	4.20	221,245	$C_{15}H_{14}O_6$	Epicatechin	[10], (std)
20.62	457.07903	3.05	125,169,305	$C_{22}H_{18}O_{11}$	Epigallocatechin gallate	[3],***
26.91	479.08408	2.01	316	$C_{21}H_{20}O_{13}$	Myricetin 3-O-galactoside	[1], (std)
32.31	609.14821	3.44	300,463	$C_{27}H_{30}O_{16}$	Quercetin rutoside	[10], (std)
33.46	463.09001	3.90	300	$C_{21}H_{20}O_{12}$	Quercetin glucoside	[10], (std)
39.29	447.09399	1.57	300	$C_{21}H_{20}O_{11}$	Quercetin rhamnoside	[10], (std)

Quantification was based on calibration curve for: *gallic acid, **dihydroxybenzoic acid, ***catechin, ****caffeic acid, std– standard

1. D'Urso, G.; Montoro, P.; Piacente, S. Detection and Comparison of Phenolic Compounds in Different Extracts of Black Currant Leaves by Liquid Chromatography Coupled with High-Resolution ESI-LTQ-Orbitrap MS and High-Sensitivity ESI-Qtrap MS. *J. Pharm. Biomed. Anal.* **2020**, *179*, doi:10.1016/j.jpba.2019.112926.

2. Zhao, Y.; Lu, H.; Wang, Q.; Liu, H.; Shen, H.; Xu, W.; Ge, J.; He, D. Rapid Qualitative Profiling and Quantitative Analysis of Phenolics in Ribes Meyeri Leaves and Their Antioxidant and Antidiabetic Activities by HPLC-QTOF-MS/MS and UHPLC-MS/MS. *J. Sep. Sci.* **2021**, *44*, 1404–1420, doi:10.1002/jssc.202000962.

3. Romani, A.; Campo, M.; Pinelli, P. HPLC/DAD/ESI-MS Analyses and Anti-Radical Activity of Hydrolyzable Tannins from Different Vegetal Species. *Food Chem.* **2012**, *130*, 214–221, doi:10.1016/j.foodchem.2011.07.009.

4. Anttonen, M.J.; Karjalainen, R.O. High-Performance Liquid Chromatography Analysis of Black Currant (Ribes Nigrum L.) Fruit Phenolics Grown Either Conventionally or Organically. *J. Agric. Food Chem.* **2006**, *54*, 7530–7538, doi:10.1021/jf0615350.

5. Xianli Wu, Liwei Gu, Ronald L. Prior, S.M. Characterization of Anthocyanins and Proanthocyanidins in Some Cultivars of Ribes , Aronia , and Sambucus and Their Antioxidant Capacity. *J. Agric. Food Chem.* **2004**, *52*, 7846–7856.

6. Efenberger-Szmechtyk, M.; Nowak, A.; Czyżowska, A.; Kucharska, A.Z.; Fecka, I. Composition and Antibacterial Activity of Aronia Melanocarpa (Michx.) Elliot, Cornus Mas L. And Chaenomeles Superba Lindl. Leaf Extracts. *Molecules* **2020**, *25*, 1–21, doi:10.3390/molecules25092011.

7. Lee, J.E.; Kim, G.S.; Park, S.; Kim, Y.H.; Kim, M.B.; Lee, W.S.; Jeong, S.W.; Lee, S.J.; Jin, J.S.; Shin, S.C. Determination of Chokeberry (Aronia Melanocarpa) Polyphenol Components Using Liquid Chromatography-Tandem Mass Spectrometry: Overall Contribution to Antioxidant Activity. *Food Chem.* **2014**, *146*, 1–5, doi:10.1016/j.foodchem.2013.09.029.

8. Cebulak, T.; Oszmiański, J.; Kapusta, I.; Lachowicz, S. Effect of UV-C Radiation, Ultra-Sonication Electromagnetic Field and Microwaves on Changes in Polyphenolic Compounds in Chokeberry (Aronia Melanocarpa). *Molecules* **2017**, *22*, doi:10.3390/molecules22071161.

9. Ossipov, V.; Zubova, M.; Nechaeva, T.; Zagoskina, N.; Salminen, J.P. The Regulating Effect of Light on the Content of Flavan-3-Ols and Derivatives of Hydroxybenzoic Acids in the Callus Culture of the Tea Plant, Camellia Sinensis L. *Biochem. Syst. Ecol.* **2022**, *101*, 104383, doi:10.1016/j.bse.2022.104383.

10. Bujor, O.C.; Le Bourvellec, C.; Volf, I.; Popa, V.I.; Dufour, C. Seasonal Variations of the Phenolic Constituents in Bilberry (Vaccinium Myrtillus L.) Leaves, Stems and Fruits, and Their Antioxidant Activity. *Food Chem.* **2016**, *213*, 58–68, doi:10.1016/j.foodchem.2016.06.042.

11. Stein-Chisholm, R.E.; Beaulieu, J.C.; Grimm, C.C.; Lloyd, S.W. Lc–Ms/Ms and Uplc–Uv Evaluation of Anthocyanins and Anthocyanidins during Rabbiteye Blueberry Juice Processing. *Beverages* **2017**, *3*, doi:10.3390/beverages3040056.

12. Bayazid, A.B.; Chun, E.M.; Al Mijan, M.; Park, S.H.; Moon, S.K.; Lim, B.O. Anthocyanins Profiling of Bilberry (Vaccinium Myrtillus L.) Extract That Elucidates Antioxidant and Anti-Inflammatory Effects. *Food Agric. Immunol.* **2021**, *32*, 713–726, doi:10.1080/09540105.2021.1986471.
